# *trans*-Diaqua­bis[5-carb­oxy-2-(3-pyrid­yl)-1*H*-imidazole-4-carboxyl­ato-κ^2^ *N* ^3^,*O* ^4^]iron(II)

**DOI:** 10.1107/S1600536809027457

**Published:** 2009-07-18

**Authors:** Wei Liu, Gang Zhang, Xia Li, Ben-Lai Wu, Hong-Yun Zhang

**Affiliations:** a Department of Chemistry and Chemical Engineering, Henan University of Urban Construction, Pingdingshan, Henan 467044, People’s Republic of China; b Department of Chemistry, Zhengzhou University, Zhengzhou, Henan 450052, People’s Republic of China

## Abstract

In the title complex, [Fe(C_10_H_6_N_3_O_4_)_2_(H_2_O)_2_], the Fe^II^ atom is located on an inversion centre and is *trans*-coordinated by two *N*,*O*-bidentate 5-carb­oxy-2-(3-pyrid­yl)-1*H*-imidazole-4-carb­oxy­l­ate ligands and two water mol­ecules, defining a distorted octa­hedral environment. A two-dimensional network of N—H⋯O and O—H⋯O hydrogen bonds extending parallel to (110) helps to stabilize the crystal packing.

## Related literature

*N*-Heterocyclic carboxylic acids are efficient N/O donors exhibiting versatile coordination modes and hydrogen bonding and can be successively deprotonated, resulting in a large diversity of supra­molecular architectures, see: Gu *et al.* (2007 ▶); Liu *et al.* (2004 ▶); Maji *et al.* (2005 ▶); Rajendiran *et al.* (2003 ▶); Sun *et al.* (2005 ▶); Zou *et al.* (2005 ▶).
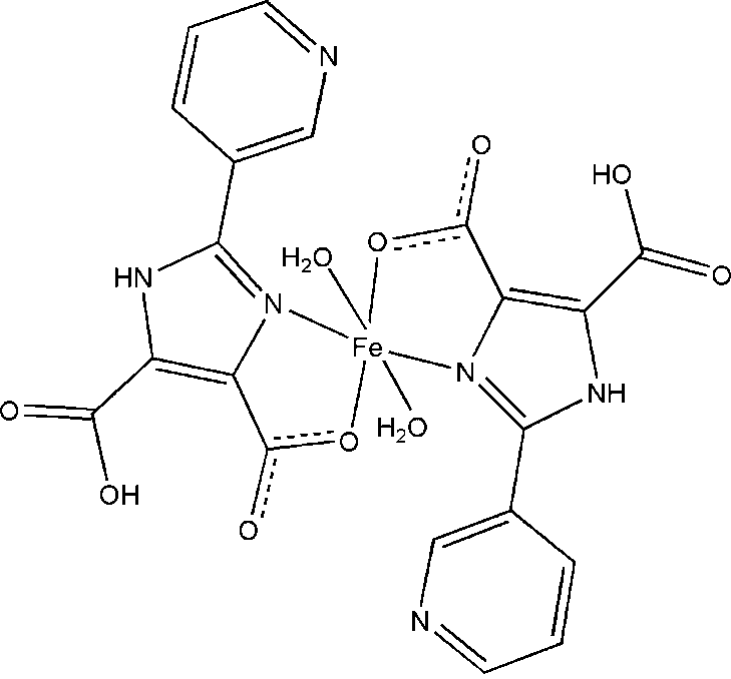


## Experimental

### 

#### Crystal data

[Fe(C_10_H_6_N_3_O_4_)_2_(H_2_O)_2_]
*M* *_r_* = 556.24
Triclinic, 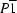

*a* = 7.0100 (14) Å
*b* = 8.6670 (17) Å
*c* = 9.4110 (19) Å
α = 82.28 (3)°
β = 83.84 (3)°
γ = 70.66 (3)°
*V* = 533.41 (18) Å^3^
*Z* = 1
Mo *K*α radiation
μ = 0.78 mm^−1^
*T* = 173 K
0.29 × 0.24 × 0.19 mm

#### Data collection

Rigaku Mercury CCD diffractometer
Absorption correction: multi-scan (*CrystalClear*; Rigaku, 2000 ▶) *T*_min_ = 0.826, *T*_max_ = 0.890
4126 measured reflections
2084 independent reflections
1693 reflections with *I* > 2σ(*I*)
*R*_int_ = 0.023

#### Refinement

*R*[*F* ^2^ > 2σ(*F* ^2^)] = 0.038
*wR*(*F* ^2^) = 0.105
*S* = 1.04
2084 reflections
172 parameters
1 restraint
H atoms treated by a mixture of independent and constrained refinement
Δρ_max_ = 0.47 e Å^−3^
Δρ_min_ = −0.55 e Å^−3^

### 

Data collection: *CrystalClear* (Rigaku, 2000 ▶); cell refinement: *CrystalClear*; data reduction: *CrystalClear*; program(s) used to solve structure: *SHELXS97* (Sheldrick, 2008 ▶); program(s) used to refine structure: *SHELXL97* (Sheldrick, 2008 ▶); molecular graphics: *SHELXTL* (Sheldrick, 2008 ▶); software used to prepare material for publication: *SHELXTL*.

## Supplementary Material

Crystal structure: contains datablocks I, global. DOI: 10.1107/S1600536809027457/bv2118sup1.cif

Structure factors: contains datablocks I. DOI: 10.1107/S1600536809027457/bv2118Isup2.hkl

Additional supplementary materials:  crystallographic information; 3D view; checkCIF report

## Figures and Tables

**Table 1:** Hydrogen-bond geometry (Å, °)

*D*—H⋯*A*	*D*—H	H⋯*A*	*D*⋯*A*	*D*—H⋯*A*
N2—H2*A*⋯O4^i^	0.83	2.09	2.852 (3)	152
O5—H5*A*⋯O3^ii^	0.89	2.03	2.899 (3)	165
O5—H5*B*⋯N3^iii^	0.84	2.01	2.780 (3)	152
O3—H3*A*⋯O2	0.868 (10)	1.613 (11)	2.479 (3)	176 (3)

Symmetry codes: (i) 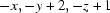; (ii) 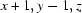; (iii) 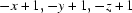.

**Fig. 1.**
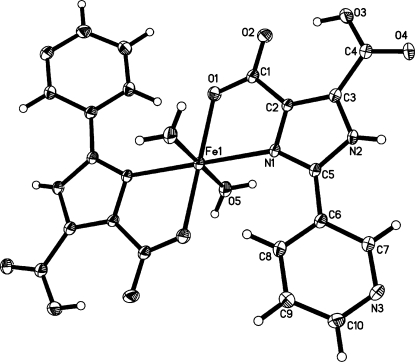
A view of the molecular of (I), showing the atom-labelling scheme and displacement ellipsoids at the 30% probability level.

**Fig. 2.**
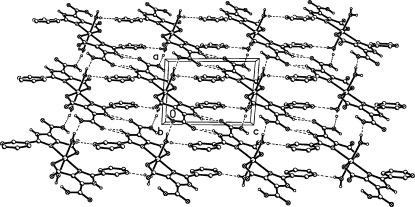
The crystal packing of (I), showing the two-dimensional hydrogen-bonding network, H atoms not involved in hydrogen bonding have been omited.

**Fig. 3.**
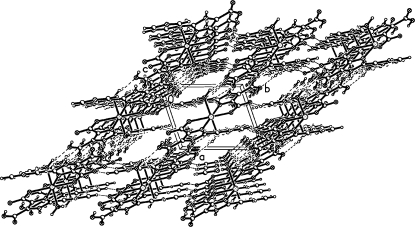
Packing diagram of two-dimensional layers along the b axis.

## supplementary crystallographic information

### Comment

N-Heterocyclic carboxylic acids, such as imidazole-4,5-dicarboxylic acid (H_3_IDC), are recognized as efficient N/O donors exhibiting versatile coordination modes and hydrogen bonding. It can be successively deprotonated to generate various species with different proton numbers (H_2_IDC^-^, HIDC^2-^, and IDC^3-^), and hence may result in a large diversity of supramolecular architectures (Sun *et al.*, 2005; Maji *et al.*, 2005; Liu *et al.*, 2004; Zou *et al.*, 2005; Rajendiran *et al.*, 2003; Gu *et al.*, 2007). In contrast to the well studied H_3_IDC, 2-(pyridin-4-yl)-1*H*-imidazole-4,5-dicarboxylate acid (H_3_PIDC), a very close analogue of H_3_IDC, still remains unexplored till now. We report here the single-crystal structure of a new compound, [Fe(H_2_PIDC)_2_(H_2_O)_2_] (I), in which the H_2_PIDC monoanion coordinates to the Fe atom, acting as a bidentate ligand.

As shown in Fig. 1, the molecule of (I) is a discrete neutral monomer, in which the Fe atom resides on a crystallographic inversion centre and the asymmetric unit contains one-half of the [Fe(H_2_PIDC)_2_(H_2_O)_2_] formula unit. Each Fe atom is six-coordinated by two N and two O atoms from two H_2_PIDC ligands and two water molecule in a highly distorted octahedral geometry. In this complex, the carboxylic acid (H_3_PIDC) ligand is singly deprotonated and bears a formal charge of -1, and the uncoordinated carboxylate atoms O3 and O2 form an intramolecular hydrogen bond (Table 1). All non-H atoms in the imidazole-4,5-dicarboxyl group are nearly coplanar [the mean deviation is 0.031 (9) Å], and the dihedral angle between imidazole group and pyridine group is 22.6 (1) °. At list in Table 2, a two-dimensional supramolecular layer is constructed *via* hydrogen-bonding interactions involving the coordinated water molecules, the carboxy O atoms and the protonated imidazole N atoms (Fig.2), and these two-dimensional layers are parallel arranged along *b* axis (Fig. 3).

### Experimental

A mixture of Fe(II) sulfate (0.056 g, 0.2 mmol), 2-(pyridin-3-yl)-1*H*-imidazole-4,5-dicarboxylic acid (0.047 g, 0.2 mmol) and water (10 ml) was sealed into a Teflon-lined stainless autoclave and heated at 433 K for 4 days. The bomb was allowed to cooled to room temperature gradually and red prismatic crystals of (I) were obtained. Analysis calculated for C_20_H_16_FeN_6_O_10_: C 43.19, H 2.90, N 15.11; found: C 43.12, H 2.94, N 15.13.

### Refinement

Water H atoms and the carboxylic acid H atom were located from difference maps and refined with a *DFIX* restraint of 0.86 (2) Å applied to the three O—H distances. Aromatic H atoms were positioned geometrically with C—H = 0.95 Å and constrained to ride on their parent atoms with *U*_iso_(H) = 1.2*U*_eq_(C).

### Crystal data

**Table:**

[Fe(C_10_H_6_N_3_O_4_)_2_(H_2_O)_2_]	*Z* = 1
*M**_r_* = 556.24	*F*(000) = 284
Triclinic, *P*1	*D*_x_ = 1.732 Mg m^−^^3^
*a* = 7.0100 (14) Å	Mo *K*α radiation, λ = 0.71073 Å
*b* = 8.6670 (17) Å	θ = 2.5–28.0°
*c* = 9.4110 (19) Å	µ = 0.78 mm^−^^1^
α = 82.28 (3)°	*T* = 173 K
β = 83.84 (3)°	Block, yellow
γ = 70.66 (3)°	0.28 × 0.24 × 0.19 mm
*V* = 533.41 (18) Å^3^	

### Data collection

**Table:**

Rigaku Mercury CCD diffractometer	2084 independent reflections
Radiation source: fine-focus sealed tube	1693 reflections with *I* > 2σ(*I*)
graphite	*R*_int_ = 0.023
ω scans	θ_max_ = 26.0°, θ_min_ = 2.5°
Absorption correction: multi-scan (*CrystalClear*; Rigaku, 2000)	*h* = −8→8
*T*_min_ = 0.826, *T*_max_ = 0.890	*k* = −10→10
4126 measured reflections	*l* = −11→11

### Refinement

**Table:**

Refinement on *F*^2^	Primary atom site location: structure-invariant direct methods
Least-squares matrix: full	Secondary atom site location: difference Fourier map
*R*[*F*^2^ > 2σ(*F*^2^)] = 0.038	Hydrogen site location: inferred from neighbouring sites
*wR*(*F*^2^) = 0.105	H atoms treated by a mixture of independent and constrained refinement
*S* = 1.04	*w* = 1/[σ^2^(*F*_o_^2^) + (0.0571*P*)^2^ + 0.2007*P*] where *P* = (*F*_o_^2^ + 2*F*_c_^2^)/3
2084 reflections	(Δ/σ)_max_ < 0.001
172 parameters	Δρ_max_ = 0.47 e Å^−^^3^
1 restraint	Δρ_min_ = −0.55 e Å^−^^3^

### Special details

**Table:**

Geometry. All e.s.d.'s (except the e.s.d. in the dihedral angle between two l.s. planes) are estimated using the full covariance matrix. The cell e.s.d.'s are taken into account individually in the estimation of e.s.d.'s in distances, angles and torsion angles; correlations between e.s.d.'s in cell parameters are only used when they are defined by crystal symmetry. An approximate (isotropic) treatment of cell e.s.d.'s is used for estimating e.s.d.'s involving l.s. planes.
Refinement. Refinement of *F*^2^ against ALL reflections. The weighted *R*-factor *wR* and goodness of fit *S* are based on *F*^2^, conventional *R*-factors *R* are based on *F*, with *F* set to zero for negative *F*^2^. The threshold expression of *F*^2^ > σ(*F*^2^) is used only for calculating *R*-factors(gt) *etc*. and is not relevant to the choice of reflections for refinement. *R*-factors based on *F*^2^ are statistically about twice as large as those based on *F*, and *R*- factors based on ALL data will be even larger.

### Fractional atomic coordinates and isotropic or equivalent isotropic displacement parameters (Å^2^)

**Table:**

	*x*	*y*	*z*	*U*_iso_*/*U*_eq_	
Fe1	0.5000	0.5000	0.0000	0.03010 (18)	
O1	0.4346 (3)	0.7540 (2)	−0.09416 (18)	0.0407 (5)	
O2	0.2752 (3)	1.0178 (2)	−0.06408 (18)	0.0410 (5)	
O3	0.0627 (3)	1.1808 (2)	0.12660 (19)	0.0408 (5)	
O4	−0.0530 (3)	1.1320 (2)	0.35321 (19)	0.0466 (5)	
O5	0.7690 (3)	0.5052 (2)	0.07395 (18)	0.0406 (5)	
N1	0.3184 (3)	0.6478 (2)	0.17970 (19)	0.0267 (4)	
N2	0.1534 (3)	0.7958 (2)	0.3559 (2)	0.0280 (5)	
H2A	0.0841	0.8214	0.4319	0.034*	
N3	0.2740 (3)	0.3678 (3)	0.6641 (2)	0.0357 (5)	
C1	0.3294 (4)	0.8639 (3)	−0.0195 (2)	0.0313 (6)	
C2	0.2649 (3)	0.8126 (3)	0.1308 (2)	0.0258 (5)	
C3	0.1588 (4)	0.9068 (3)	0.2385 (2)	0.0277 (5)	
C4	0.0479 (4)	1.0858 (3)	0.2420 (3)	0.0322 (6)	
C5	0.2483 (3)	0.6410 (3)	0.3177 (2)	0.0259 (5)	
C6	0.2686 (4)	0.4921 (3)	0.4180 (2)	0.0269 (5)	
C7	0.2554 (4)	0.4991 (3)	0.5672 (2)	0.0313 (6)	
H3	0.2321	0.6026	0.6011	0.038*	
C8	0.3003 (4)	0.3402 (3)	0.3698 (3)	0.0332 (6)	
H4	0.3060	0.3303	0.2700	0.040*	
C9	0.3234 (4)	0.2031 (3)	0.4700 (3)	0.0379 (6)	
H1	0.3486	0.0977	0.4392	0.045*	
C10	0.3092 (4)	0.2212 (3)	0.6157 (3)	0.0360 (6)	
H2	0.3250	0.1268	0.6833	0.043*	
H5A	0.8761	0.4145	0.0835	0.043*	
H5B	0.7552	0.5726	0.1339	0.043*	
H3A	0.137 (4)	1.128 (3)	0.057 (2)	0.043*	

### Atomic displacement parameters (Å^2^)

**Table:**

	*U*^11^	*U*^22^	*U*^33^	*U*^12^	*U*^13^	*U*^23^
Fe1	0.0395 (3)	0.0268 (3)	0.0151 (3)	0.0007 (2)	0.00222 (19)	−0.00396 (19)
O1	0.0548 (12)	0.0334 (10)	0.0192 (8)	0.0012 (9)	0.0110 (8)	−0.0027 (7)
O2	0.0584 (12)	0.0293 (10)	0.0224 (9)	−0.0029 (9)	0.0075 (8)	0.0046 (7)
O3	0.0584 (13)	0.0267 (10)	0.0264 (10)	−0.0017 (9)	0.0053 (8)	−0.0029 (7)
O4	0.0665 (14)	0.0351 (10)	0.0239 (9)	0.0033 (9)	0.0048 (9)	−0.0113 (8)
O5	0.0457 (11)	0.0410 (11)	0.0269 (9)	0.0002 (9)	−0.0032 (8)	−0.0112 (8)
N1	0.0350 (11)	0.0254 (10)	0.0140 (9)	−0.0028 (9)	0.0027 (8)	−0.0035 (8)
N2	0.0352 (11)	0.0279 (11)	0.0152 (9)	−0.0039 (9)	0.0064 (8)	−0.0052 (8)
N3	0.0473 (13)	0.0365 (12)	0.0190 (10)	−0.0098 (10)	0.0000 (9)	0.0003 (9)
C1	0.0391 (14)	0.0307 (13)	0.0164 (11)	−0.0024 (11)	0.0023 (10)	−0.0019 (10)
C2	0.0318 (12)	0.0249 (12)	0.0145 (11)	−0.0025 (10)	0.0024 (9)	−0.0013 (9)
C3	0.0342 (13)	0.0276 (13)	0.0182 (11)	−0.0059 (10)	0.0001 (9)	−0.0030 (9)
C4	0.0385 (14)	0.0304 (13)	0.0225 (12)	−0.0032 (11)	−0.0022 (10)	−0.0054 (10)
C5	0.0304 (12)	0.0266 (12)	0.0165 (11)	−0.0038 (10)	0.0008 (9)	−0.0039 (9)
C6	0.0303 (12)	0.0290 (13)	0.0181 (11)	−0.0064 (10)	0.0021 (9)	−0.0020 (9)
C7	0.0419 (15)	0.0288 (13)	0.0204 (12)	−0.0075 (11)	0.0001 (10)	−0.0046 (10)
C8	0.0447 (15)	0.0312 (14)	0.0198 (11)	−0.0080 (11)	0.0048 (10)	−0.0051 (10)
C9	0.0508 (17)	0.0270 (13)	0.0319 (14)	−0.0079 (12)	0.0008 (12)	−0.0043 (11)
C10	0.0435 (15)	0.0301 (14)	0.0285 (13)	−0.0075 (12)	0.0012 (11)	0.0032 (11)

### Geometric parameters (Å, °)

**Table:**

Fe1—O5^i^	2.095 (2)	N2—C3	1.369 (3)
Fe1—O5	2.095 (2)	N2—H2A	0.8325
Fe1—O1^i^	2.1764 (19)	N3—C7	1.338 (3)
Fe1—O1	2.1764 (19)	N3—C10	1.344 (3)
Fe1—N1	2.2719 (19)	C1—C2	1.493 (3)
Fe1—N1^i^	2.2719 (19)	C2—C3	1.380 (3)
O1—C1	1.244 (3)	C3—C4	1.490 (3)
O2—C1	1.284 (3)	C5—C6	1.469 (3)
O3—C4	1.289 (3)	C6—C8	1.390 (3)
O3—H3A	0.868 (10)	C6—C7	1.405 (3)
O4—C4	1.234 (3)	C7—H3	0.9500
O5—H5A	0.8910	C8—C9	1.390 (3)
O5—H5B	0.8412	C8—H4	0.9500
N1—C5	1.339 (3)	C9—C10	1.390 (4)
N1—C2	1.378 (3)	C9—H1	0.9500
N2—C5	1.364 (3)	C10—H2	0.9500
			
O5^i^—Fe1—O5	180.0	O2—C1—C2	118.6 (2)
O5^i^—Fe1—O1^i^	90.33 (8)	N1—C2—C3	110.46 (19)
O5—Fe1—O1^i^	89.67 (8)	N1—C2—C1	119.4 (2)
O5^i^—Fe1—O1	89.67 (8)	C3—C2—C1	130.1 (2)
O5—Fe1—O1	90.33 (8)	N2—C3—C2	105.0 (2)
O1^i^—Fe1—O1	180.00 (9)	N2—C3—C4	121.4 (2)
O5^i^—Fe1—N1	89.91 (7)	C2—C3—C4	133.4 (2)
O5—Fe1—N1	90.09 (7)	O4—C4—O3	124.8 (2)
O1^i^—Fe1—N1	103.66 (7)	O4—C4—C3	118.2 (2)
O1—Fe1—N1	76.34 (7)	O3—C4—C3	117.0 (2)
O5^i^—Fe1—N1^i^	90.09 (7)	N1—C5—N2	110.1 (2)
O5—Fe1—N1^i^	89.91 (7)	N1—C5—C6	126.8 (2)
O1^i^—Fe1—N1^i^	76.34 (7)	N2—C5—C6	123.1 (2)
O1—Fe1—N1^i^	103.66 (7)	C8—C6—C7	117.7 (2)
N1—Fe1—N1^i^	180.000 (1)	C8—C6—C5	121.7 (2)
C1—O1—Fe1	117.71 (15)	C7—C6—C5	120.7 (2)
C4—O3—H3A	113 (2)	N3—C7—C6	123.6 (2)
Fe1—O5—H5A	121.1	N3—C7—H3	118.2
Fe1—O5—H5B	115.7	C6—C7—H3	118.2
H5A—O5—H5B	115.2	C9—C8—C6	118.9 (2)
C5—N1—C2	105.66 (18)	C9—C8—H4	120.5
C5—N1—Fe1	145.46 (16)	C6—C8—H4	120.5
C2—N1—Fe1	108.78 (13)	C8—C9—C10	119.5 (2)
C5—N2—C3	108.75 (19)	C8—C9—H1	120.2
C5—N2—H2A	126.9	C10—C9—H1	120.2
C3—N2—H2A	123.4	N3—C10—C9	122.2 (2)
C7—N3—C10	118.1 (2)	N3—C10—H2	118.9
O1—C1—O2	123.7 (2)	C9—C10—H2	118.9
O1—C1—C2	117.7 (2)		
			
O5^i^—Fe1—O1—C1	87.8 (2)	N1—C2—C3—N2	1.7 (3)
O5—Fe1—O1—C1	−92.2 (2)	C1—C2—C3—N2	−176.4 (3)
O1^i^—Fe1—O1—C1	−157 (100)	N1—C2—C3—C4	−172.4 (3)
N1—Fe1—O1—C1	−2.2 (2)	C1—C2—C3—C4	9.5 (5)
N1^i^—Fe1—O1—C1	177.8 (2)	N2—C3—C4—O4	−1.3 (4)
O5^i^—Fe1—N1—C5	95.8 (3)	C2—C3—C4—O4	172.0 (3)
O5—Fe1—N1—C5	−84.2 (3)	N2—C3—C4—O3	179.7 (2)
O1^i^—Fe1—N1—C5	5.5 (3)	C2—C3—C4—O3	−7.0 (4)
O1—Fe1—N1—C5	−174.5 (3)	C2—N1—C5—N2	0.0 (3)
N1^i^—Fe1—N1—C5	−141 (100)	Fe1—N1—C5—N2	175.5 (2)
O5^i^—Fe1—N1—C2	−88.65 (16)	C2—N1—C5—C6	−179.4 (2)
O5—Fe1—N1—C2	91.35 (16)	Fe1—N1—C5—C6	−3.8 (5)
O1^i^—Fe1—N1—C2	−178.96 (15)	C3—N2—C5—N1	1.1 (3)
O1—Fe1—N1—C2	1.04 (15)	C3—N2—C5—C6	−179.5 (2)
N1^i^—Fe1—N1—C2	34 (100)	N1—C5—C6—C8	−23.2 (4)
Fe1—O1—C1—O2	−177.8 (2)	N2—C5—C6—C8	157.6 (2)
Fe1—O1—C1—C2	2.8 (3)	N1—C5—C6—C7	157.2 (2)
C5—N1—C2—C3	−1.0 (3)	N2—C5—C6—C7	−22.1 (4)
Fe1—N1—C2—C3	−178.39 (16)	C10—N3—C7—C6	0.7 (4)
C5—N1—C2—C1	177.3 (2)	C8—C6—C7—N3	0.8 (4)
Fe1—N1—C2—C1	−0.1 (3)	C5—C6—C7—N3	−179.5 (2)
O1—C1—C2—N1	−1.8 (4)	C7—C6—C8—C9	−1.9 (4)
O2—C1—C2—N1	178.8 (2)	C5—C6—C8—C9	178.4 (2)
O1—C1—C2—C3	176.1 (3)	C6—C8—C9—C10	1.6 (4)
O2—C1—C2—C3	−3.3 (4)	C7—N3—C10—C9	−1.1 (4)
C5—N2—C3—C2	−1.7 (3)	C8—C9—C10—N3	−0.1 (4)
C5—N2—C3—C4	173.3 (2)		

Symmetry codes: (i) −*x*+1, −*y*+1, −*z*.

### Hydrogen-bond geometry (Å, °)

**Table:**

*D*—H···*A*	*D*—H	H···*A*	*D*···*A*	*D*—H···*A*
N2—H2A···O4^ii^	0.83	2.09	2.852 (3)	152
O5—H5A···O3^iii^	0.89	2.03	2.899 (3)	165
O5—H5B···N3^iv^	0.84	2.01	2.780 (3)	152
O3—H3A···O2	0.87 (1)	1.61 (1)	2.479 (3)	176 (3)

Symmetry codes: (ii) −*x*, −*y*+2, −*z*+1; (iii) *x*+1, *y*−1, *z*; (iv) −*x*+1, −*y*+1, −*z*+1.
